# Feline mammary carcinoma-derived extracellular vesicle promotes liver metastasis via sphingosine kinase-1-mediated premetastatic niche formation

**DOI:** 10.1186/s42826-023-00180-5

**Published:** 2023-11-09

**Authors:** Yi-Chih Chang, Hao-Ping Liu, Hsiao-Li Chuang, Jiunn-Wang Liao, Pei-Ling Kao, Hsun-Lung Chan, Ter-Hsin Chen, Yu-Chih Wang

**Affiliations:** 1 Department of Medical Laboratory Science & Biotechnology, College of Medical and Health Science, Asia University, Taichung, Taiwan; 2 Department of Veterinary Medicine, National Chung Hsing University, Taichung, Taiwan; 3 National Laboratory Animal Center, National Applied Research Laboratories, Taipei, Taiwan; 4 Graduate Institute of Veterinary Pathobiology, National Chung Hsing University, 145 Xingda Rd., South Dist., Taichung, 40227 Taiwan; 5 Veterinary Research Institute, Ministry of Agriculture, Zhunan, Taiwan

**Keywords:** Extracellular vesicle, Sphingosine kinase-1, Pre-metastatic niche, Feline mammary carcinoma, Hepatic stellate cell

## Abstract

**Background:**

Feline mammary carcinoma (FMC) is one of the most prevalent malignancies of female cats. FMC is highly metastatic and thus leads to poor disease outcomes. Among all metastases, liver metastasis occurs in about 25% of FMC patients. However, the mechanism underlying hepatic metastasis of FMC remains largely uncharacterized.

**Results:**

Herein, we demonstrate that FMC-derived extracellular vesicles (FMC-EVs) promotes the liver metastasis of FMC by activating hepatic stellate cells (HSCs) to prime a hepatic premetastatic niche (PMN). Moreover, we provide evidence that sphingosine kinase 1 (SK1) delivered by FMC-EV was pivotal for the activation of HSC and the formation of hepatic PMN. Depletion of SK1 impaired cargo sorting in FMC-EV and the EV-potentiated HSC activation, and abolished hepatic colonization of FMC cells.

**Conclusions:**

Taken together, our findings uncover a previously uncharacterized mechanism underlying liver-metastasis of FMC and provide new insights into prognosis and treatment of this feline malignancy.

**Supplementary Information:**

The online version contains supplementary material available at 10.1186/s42826-023-00180-5.

## Background

Feline mammary carcinoma (FMC) is one of the most common malignancies of female cats [1], and the FMC patients often have short survival time ranging from 1 to 3 months [2–4]. Metastasis is the major cause of FMC-related death, and regional lymph nodes, lung, and liver are the tissues where FMC metastasis occurs most commonly [3, 5]. Among all metastases, liver metastasis takes place in about 25% of FMC patients [3]. Despite the poor prognosis of FMC attributed to metastases, the underlying mechanism remains uncharacterized and hence the improvement of treatment regimens for FMC is impeded.

In general, cancer cells accomplish a multistep cascade toward distant metastasis, including invasion, intravasation into the circulation, survival in circulation, extravasation, and adaptation to the foreign microenvironment to establish a distal tumor locus [6]. The main rate-determining step for metastasis cascade occurs during the colonization of tumor cells in distant sites [6, 7]. Primary tumor cells can modulate the formation of a premetastatic niche (PMN) in distant tissues via the release of tumor-derived factors and extracellular vesicles (EVs). The tumor-derived EVs contribute to the restructuring of the PMN that provides a supportive and receptive microenvironment for dissemination of tumor cells, which involves angiogenesis, extracellular matrix (ECM) remodeling, and immunosuppression for promoting tumor metastasis [8–10].

Hepatic stellate cells (HSCs) are postulated as an important cellular component in liver PMN formation. Active HSC promotes incoming cancer cell survival and growth in the liver microenvironment by supplying cancer cells with growth factors and cytokines, regulating ECM turnover, and suppressing immune response [7, 11, 12]. The phenotypic changes of active HSCs include expression of α-smooth muscle actin (α-SMA), induction of cell proliferation, and increase in ECM deposits [7, 13]. Moreover, sphingosine kinase 1 (SK1) has been demonstrated to be involved in the regulation of HSC activation in liver-related diseases [14–16]. SK1 phosphorylates sphingosine to form sphingosine-1-phosphate (S1P) which in turn binds to the S1P receptor (S1PR). Notably, SK1-overexpressing EVs derived from endothelial cells can regulate HSC signaling and migration through a SK1/S1P/S1PR2 signaling cascade [17]. In addition, the SK1/S1P/S1PR signaling axis also plays crucial roles in HSC activation and liver fibrosis [18]. Furthermore, hepatocytes induce release of serum amyloid A via Interleukin 6 (IL-6)-mediated Signal transducer and activator of transcription 3 (STAT3) activation to promote the establishment of a hepatic PMN which facilitates liver metastasis of pancreatic cancer in a xenograft mouse model [19]. Since IL-6/STAT3-mediated signaling pathways are downstream targets of S1PR signaling [20–22], it is implicated that SK1-transduced signaling in HSCs is vital for establishment of hepatic PMN.

Despite the reported roles of SK1 in cancer progression and metastasis, the function of SK1 in FMC remains largely unknown. In this study, we demonstrated that SK1-expressing FMCs activated HSCs via release of SK1-containing EVs to establish a hepatic PMN which facilitated FMC metastasis to the liver. Importantly, treatment with an SK1 inhibitor or depletion of SK1 significantly attenuated the EV-potentiated HSC activation and concomitant liver metastasis of FMC. Taken together, our findings uncover a previously uncharacterized mechanism underlying liver-metastasis of FMC, providing new insights into clinical prognosis and treatment of this feline malignancy.

## Results

### Isolation and characterization of FMC-EV

**Fig. 1:**
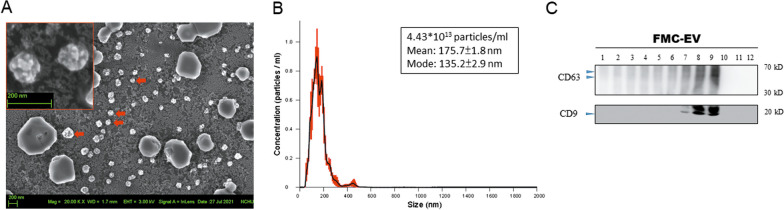
Characterization of properties of FMC-EV. **A** Isolated FMC-EV (red arrowhead) observed under a scanning electron microscope. The inset is a magnified image. Arrowhead indicates EVs. **B** Nanoparticle tracking analysis of the isolated FMC-EV. **C** Analysis of FMC-EV using size-exclusion chromatography. The resulting fractions were analyzed by Western blotting with indicated antibodies

We isolated FMC-1807 cell-derived EVs (FMC-EVs) to decipher their effects on FMC metastasis. The morphology of isolated EV was observed using scanning electron microscopy, as shown in Fig. 1A. Nanoparticle tracking analysis demonstrated that the mean diameter of FMC-EV was 175.5 nm, ranging from 50 to 200 nm (Fig. 1B). Size-exclusion chromatography further revealed that FMC-EV was enriched in fractions 8 and 9, where the EV markers CD9 and CD63 were detected by Western blotting analysis (Fig. 1C).

### FMC-EV promoted the liver metastasis of FMC

**Fig. 2:**
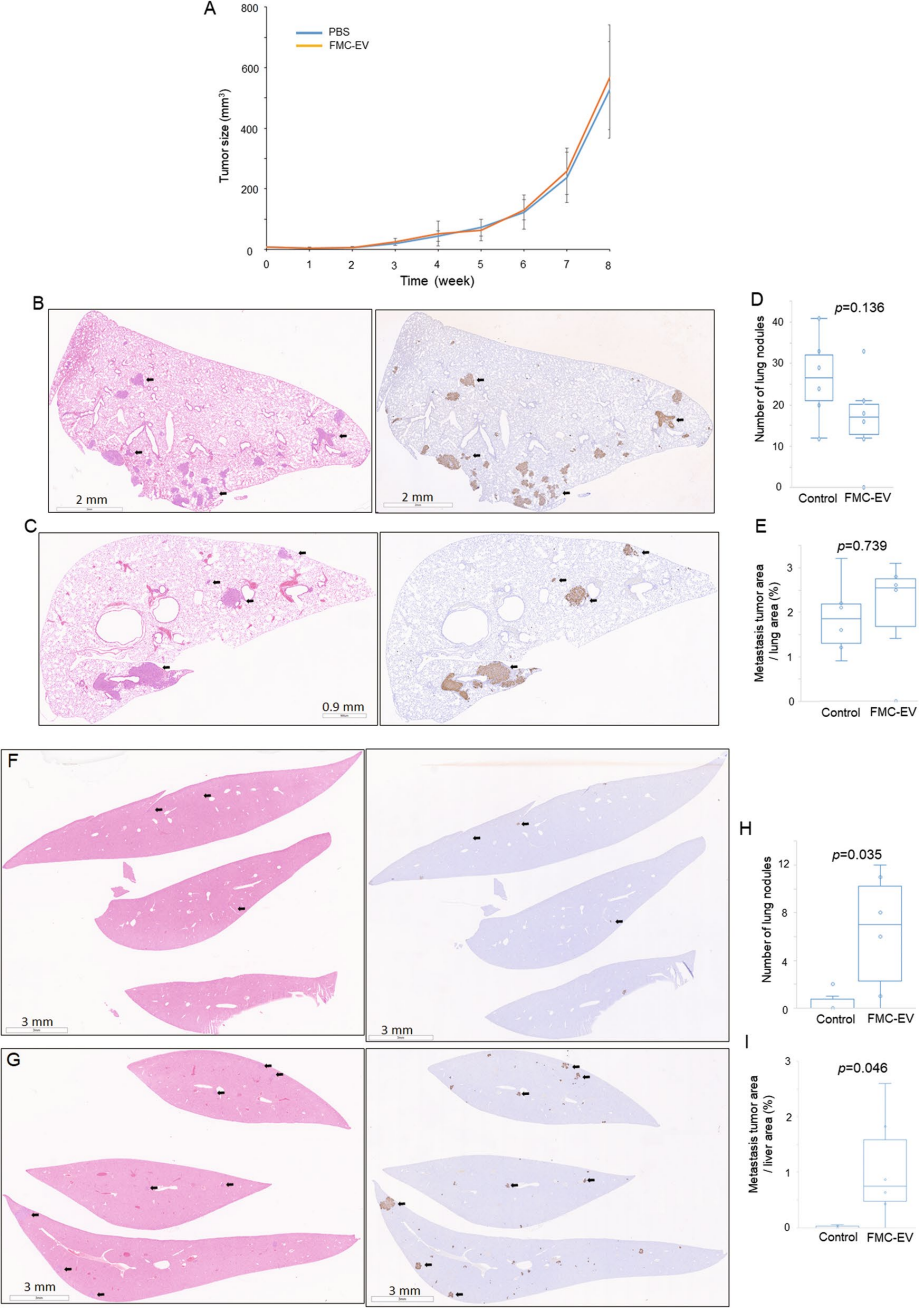
FMC-EV enhanced liver metastasis of FMC in FMC-xenograft-bearing mice. **A** Growth of FMC-1807-xenograft in mice injected with FMC-EV or PBS was measured weekly with a caliper. *n* = 6 in each group. **B**, **C** H&E staining (left panel) of the lung taken from a FMC-bearing mouse injected with PBS (**B**) or with FMC-EV (**C**). Vimentin expression (brown) in metastasized FMC in lung was analyzed by IHC (right panel). **D** The FMC foci in the lung were quantified by counting the number of foci which were vimentin-positive and presented as a ratio of the area of FMC foci to that of the entire lung section (**E**). **F**, **G** H&E staining (left panel) of the liver taken from an FMC-bearing mouse injected with PBS (**F**) or with FMC-EV (**G**). Vimentin expression in metastasized FMC in liver was analyzed by IHC (right panel). **H** The FMC foci in the liver were quantified by counting the number of foci which were vimentin-positive and presented a ratio of the area of FMC foci to that of the entire liver section (**I**). The statistical significance between groups was determined using the Student’s t-test

To elucidate the effect of FMC-EV on FMC growth and progression in vivo, we used an orthotopic patient-derived-xenograft (PDX) model of highly metastatic FMC-1807 transplanted into the mammary fat pads of female NPG mice. Three weeks post-transplantation, the mice were intraperitoneally injected with FMC-EV or PBS weekly for five weeks and subsequently sacrificed to observe the affected tissues. Results showed that the primary tumor load was not significantly changed in mice injected with FMC-EV compared with PBS-injected controls (Fig. 2A). Histologic examination and IHC analysis of vimentin expression in lung and liver sections revealed that FMC-EV had little effect on lung metastasis of FMC (Fig. 2C vs. B) either in the number (Fig. 2D) or in the area of tumor foci (Fig. 2E). In contrast, FMC-EV significantly enhanced hepatic metastasis of FMC (Fig. 2G vs. 2F) in both the number (Fig. 2H) and the area of tumor foci (Fig. 2I). These results suggest that FMC-EV selectively accelerates liver metastasis of FMC without affecting the growth of primary tumor.

### Selective uptake of FMC-EV in FMC cells, HSCs, and hepatocytes

**Fig. 3:**
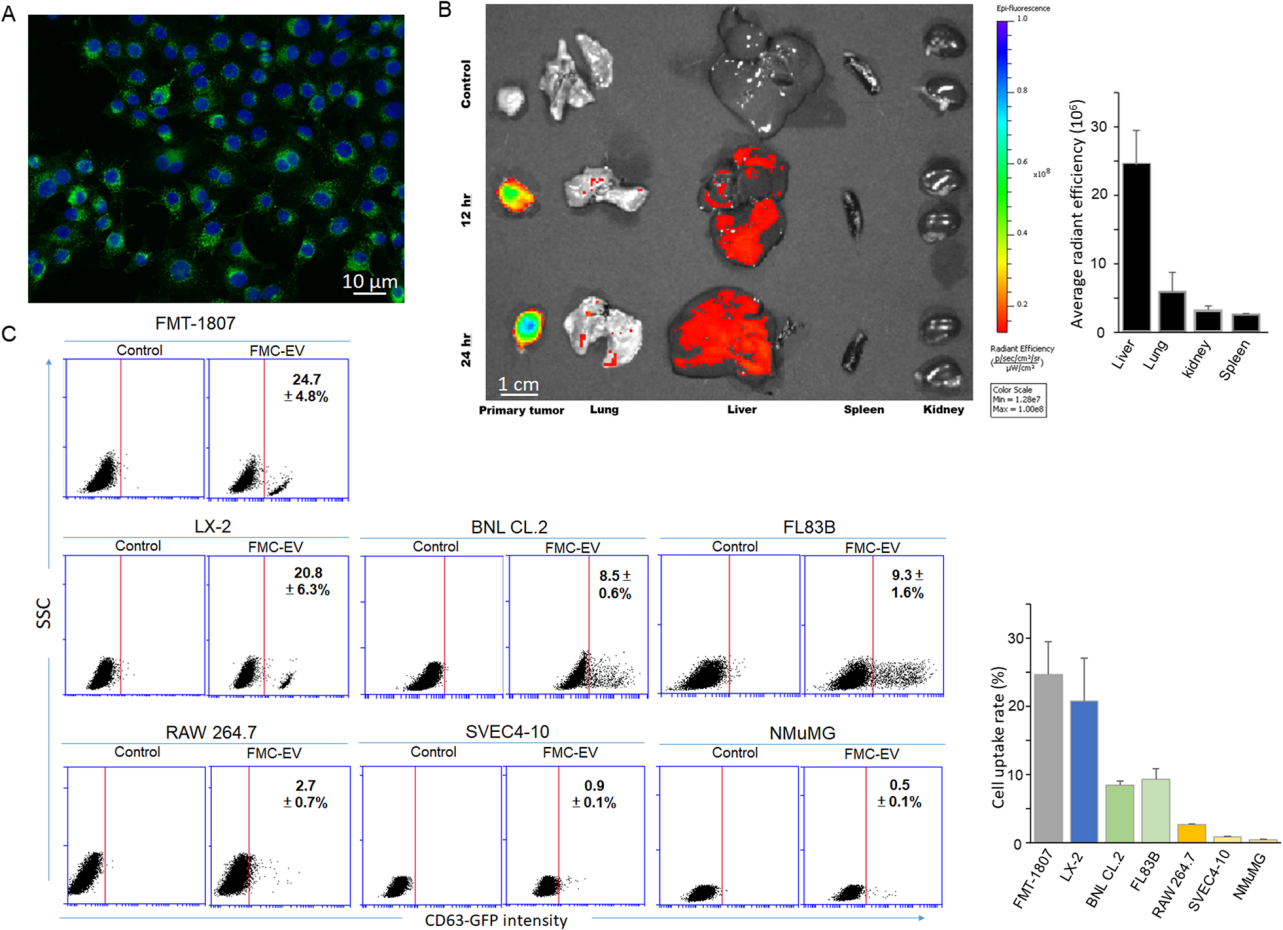
Uptake of FMC-EV expressing CD63-GFP in vivo and in vitro. **A** Observation of CD63-GFP expression in vesicles in CD63-GFP-expressing FMC-1807 cells by epifluorescence microscopy. Bar, 10 μm. **B** Representative IVIS-imaging data of the primary tumor, lung, liver, spleen, and kidneys taken from FMC-xenograft-bearing mice, which had been intraperitoneally injected with CD63-GFP-expresing EV or PBS (control) 12 or 24 h earlier. **C** Flow cytometry analysis of uptake of CD63-GFP-expressing EV in various types of cells. FMC-1807, LX-2 (human hepatic stellate cell, HSC), BNL CL.2 and FL83B (mouse hepatocyte), RAW 264.7 (mouse macrophage), SVEC4-10 (mouse endothelial cell), and NMuMG (mouse mammary epithelial cell) were incubated with CD63-GFP-expressing EV (1 × 10^9^ EV particles/1 × 10^4^ cells) for 30 min and subsequently analyzed by flow cytometry. The percentage of GFP-positive cells of each cell type (*n* = 3) was presented as the mean ± SD

We next investigated the targeting tissues of FMC-EV. To this aim, we generated CD63-GFP-expressing FMC-1807 cells (Fig. 3A) and isolated the CD63-GFP-expresing EV derived from the cells for ex vivo and in vitro assays. FMC-1807-PDX-bearing mice were intraperitoneally injected with CD63-GFP-expressing EV and sacrificed at 12- or 24-h post-injection to detect the cellular uptake of EV in multiple tissues, including the primary tumor, lung, liver, spleen and kidney. Results showed that CD63-GFP-expressing EV was most significantly ingested in the primary tumor and the liver from 12-h post-injection (Fig. 3B). Moreover, the EV uptake was also detected in a small portion of lung, in contrast to undetectable EV uptake in the spleen and kidneys.

We further examined the cell tropism of CD63-GFP-expressing EV by incubating the EV with various types of cells and subsequently analyzed the cellular uptake of EV using flow cytometry and epifluorescence microscopy (Additional file 1: Fig. S1). As shown in Fig. 3C, the percentages of FMC-1807 cells and HSC LX-2 cells which ingested CD63-GFP-expressing EVs were 24.7 ± 4.8% and 20.8 ± 6.3%, respectively, remarkably higher than those of hepatocytes BNL CL.2 and FL83B (8.5 ± 0.6% and 9.3 ± 1.6%, respectively), macrophages RAW 264.7 (2.7 ± 0.1%), endothelial cells SVEC4-10 (0.9 ± 0.1%), and mammary epithelial cells NMuMG (0.5 ± 0.1%). Results indicate that FMC-EV exhibits a propensity for cellular ingestion by FMC and HSC.

### FMC-EVs promote liver metastasis of FMC by priming a pre-metastatic niche

**Fig. 4:**
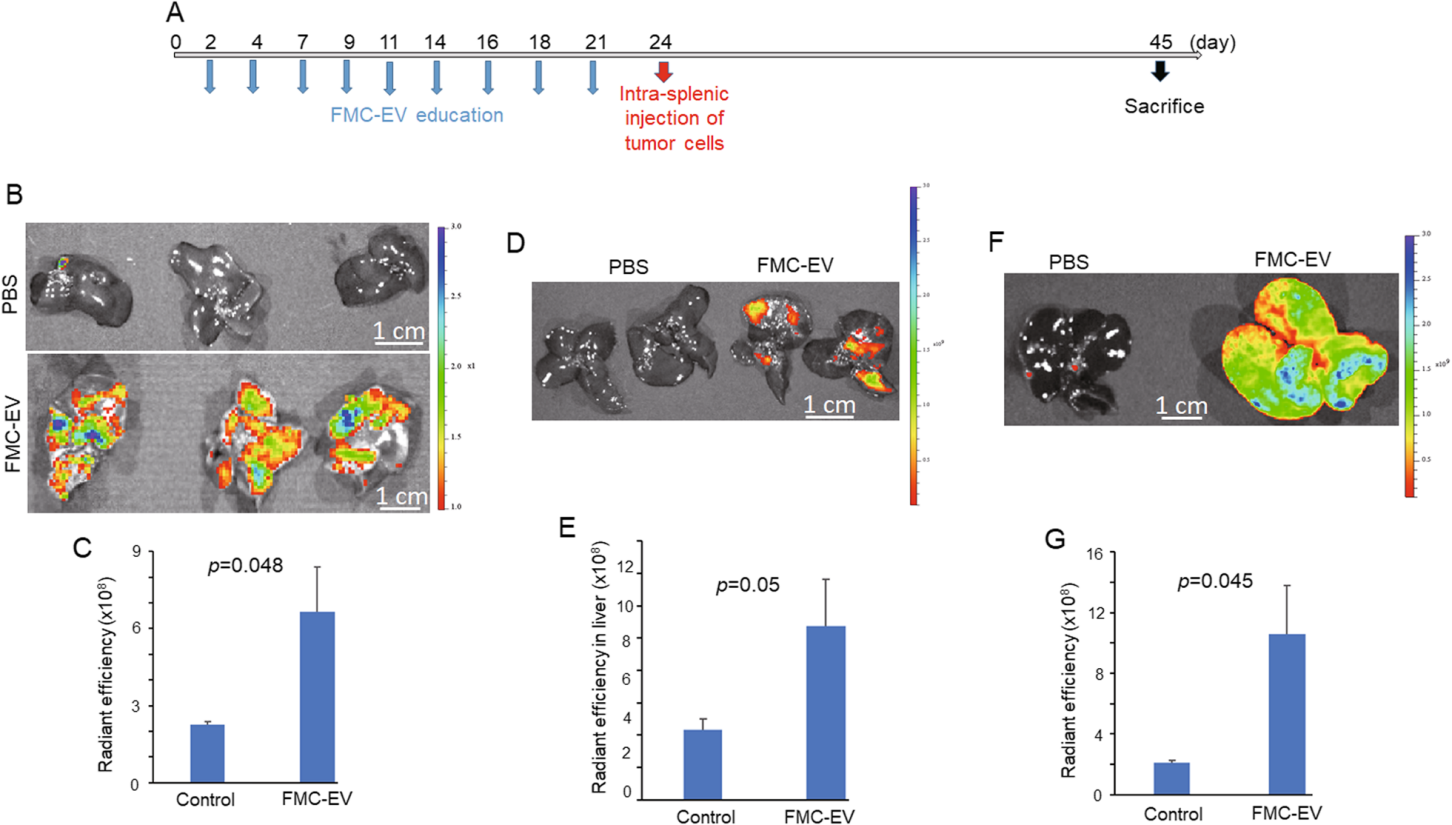
Promoting roles of FMC-EV in the formation of a pre-metastatic niche (PMN) in the liver. **A** Schematic illustration for a mouse model established to verify an EV-educated liver PMN. NPG mice were intraperitoneally injected with FMC-EV (1 × 10^10^ particles) or PBS every two or three days for nine times and then intrasplenically injected with RFP-expressing tumor cells. Livers of the mice were harvested three weeks later and subjected to IVIS imaging to detect tumor cells colorized in the liver. **B**, **D**, **F** Representative results of ex vivo imaging of livers harvested from the mice intraperitoneally injected with FMC-EVs or PBS, followed by intrasplenic injection with FMC-1807-RFP (**B**), FMC-1806-RFP (**D**), or MDA-MB-231 (**F**). **C**, **E**, **G** Fluorescence intensity of FMC-1807-RFP (**C**), FMC-1806-RFP (**E**), or MDA-MB-231 (**G**) in liver was acquired as radiant efficiency with a setting of excitation at 570 nm and emission at 620 nm. The PBS-injected group was denoted as Control. *n* = 3 in each group. Quantitation data are presented as the mean ± SD. The Student’s t-test was used to determine the statistical significance

To verify if FMC-EV primed a hepatic pre-metastatic niche (PMN), we experimentally modeled metastatic dissemination of FMC to liver by intraperitoneally injecting female NPG mice with FMC-EV or PBS triple times a week over a course of three weeks, followed by intrasplenic injection with FMC-1807-RFP, FMC-1806-RFP or MDA-MB-231 cells, as illustrated in Fig. 4A. The liver susceptibility to colonization of metastasized FMC cells was evaluated by ex vivo IVIS imaging. As shown in Fig. 4B and C, the colorization and growth of FMC-1807-RFP cells in liver pre-conditioned by FMC-EV were significantly enhanced compared with that in the PBS control. Consistent results were observed in mice injected with FMC-1806-RFP cells (Fig. 4D-E). To evaluate if the liver PMN conditioned by FMC-EV was also susceptible to metastatic dissemination of tumor cells other than FMC, we injected human breast adenocarcinoma cells MDA-MB-231-RFP into the mice after a course of injection with FMC-EV. Results showed that the tumor foci in livers of the mice injected with FMC-EV were significantly increased compared with that in the control group (Fig. 4G and F). Data collectively demonstrate that FMC-EV promotes hepatic metastasis of mammary carcinoma by conditioning a liver PMN.

### SK1 was delivered by FMC-EV and required for the activation of HSC

**Fig. 5:**
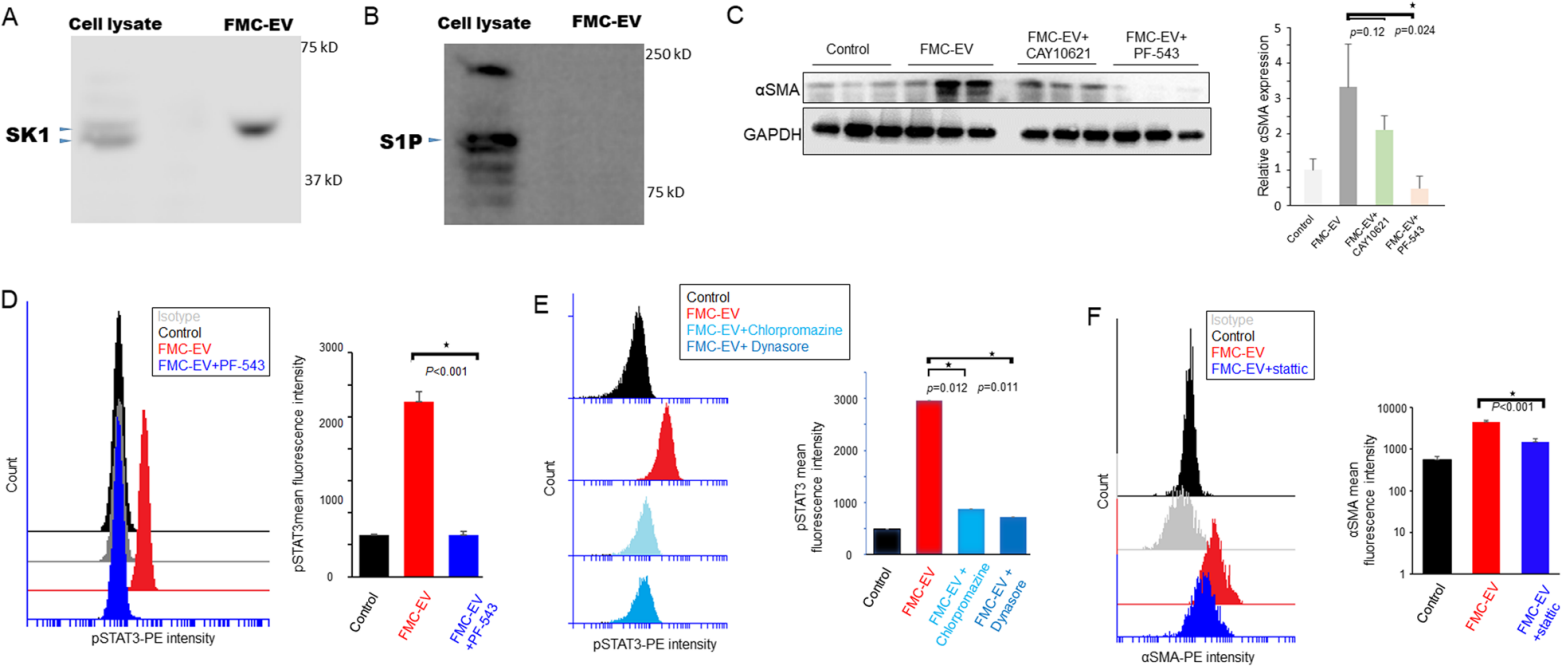
SK1 was delivered by FMC-EV and required for the EV-induced activation of HSC. **A** Detection of SK1 expression in cell lysate and EV of FMC-1807 using Western blotting. **B** Detection of S1P in cell lysate but not in EV of FMC-1807 using Western blotting. **C** SK1 inhibitors abolished activation of LX-2 cell by FMC-EV. LX-2 cells were incubated with PBS or FMC-EV in the presence or absence of a SK1 inhibitor (CAY10621 or PF-543) for 24 h. The expression of α-SMA, which serves as an activation marker, in LX-2 cells was analyzed by Western blotting. The samples were analyzed in triplicate. Protein band intensity was quantified using ImageJ (lower panel). **D** Flow cytometric analysis of the phosphorylated STAT3 (p-STAT3) level in LX-2 cells after treatment with PBS (control), FMC-EV, or FMC-EV plus PF-543 (left panel). The mean fluorescence intensity of p-STAT3 is shown (right panel). **E** Flow cytometric analysis of the p-STAT3 level of LX-2 cells after treatment with PBS (control), FMC-EV, FMC-EV plus the endocytosis inhibitor (Chlorpromazine, 10 µg/ml)) or FMC-EV plus the dynamin inhibitor (Dynasore, 10 μM) for 24 h. **F** Flow cytometric analysis of the α-SMA level of LX-2 cells after treatment with PBS (control), FMC-EV, or FMC-EV plus a STAT3 inhibitor (Stattic) for 24 h (left panel). The mean fluorescence intensity of α-SMA is shown (right panel). *n* = 3 in each group. Quantitation data are presented as the mean ± SD. The Student’s t-test was used to determine the statistical significance

We previously found that SK1 is overexpressed in FMC [23]. Notably, SK1 is reported as a component in EVs and can play a role in liver fibrosis which involves activation of HSC [14–18]. To evaluate if SK1 participated in the pro-metastatic effect of FMC-EV, we first examined whether SK1 was detected in FMC-EV by conducting Western blotting. Results showed that SK1 was present in FMC-EV (Fig. 5A). In contrast, the active SK1-generated product, S1P, was undetectable in FMC-EV (Fig. 5B). To elucidate the role of SK1 in the activation of HSC by FMC-EV, we incubated LX-2 cells with FMC-EV in the presence of SK1 inhibitors for 24 h, and harvested the cell lysates for Western blotting analysis of an HSC activation marker, α-SMA. As shown in Fig. 5C, incubation with FMC-EV significantly activated LX-2 cells as indicated by α-SMA induction, compared with the PBS-treated control. By contrast, treatment with a SK1 inhibitor CAY10621 or PF-543 remarkably repressed the EV-induced expression of α-SMA, indicating that SK1 was required for the EV-induced activation of HSC.

As STAT3 activation has been shown to mediate the formation of a hepatic PMN, we next investigated whether SK1 conferred the effect of FMC-EV through activating STAT3. LX-2 cells were incubated with FMC-EV in the presence of PF-543 (a SK1 inhibitor) and subsequently applied to flow cytometric analysis of phosphorylated STAT3, the active form of STAT3. As shown in Fig. 5D, incubation with FMC-EV significantly induced phosphorylation of STAT3 compared with the PBS-treated control. However, treatment with PF-543 significantly abolished the activation of STAT3 induced by FMC-EV, demonstrating that SK1 was required for the EV-induced STAT3 activation. We next determined whether the SK1-STAT3 axis induced by FMC-EVs in HSCs requires membrane internalization. LX-2 cells were incubated with FMC-EV in the presence of an endocytosis inhibitor (chlorpromazine) or an inhibitor of dynamin GTPase (dynasore) and subsequently subjected to flow cytometric analysis of phosphorylated STAT3. As seen in Fig. 5E, treatment of chlorpromazine or dynasore markedly attenuated the FMC-EV-induced STAT3 phosphorylation. These data indicate that FMC-EV-induced STAT3 activation in HSC requires membrane internalization.

To further verify the role of STAT3 in the FMC-EV-mediated activation of HSC, we incubated LX-2 cells with FMC-EV in the presence of a STAT3 inhibitor and subsequently examined the expression level of α-SMA. As shown in Fig. 5F, treatment with a STAT3 inhibitor (Stattic) remarkably repressed the induction of α-SMA expression by FMC-EV. Taken together, results reveal that the FMC-derived EV exerts activation of HSC by transducing a SK1-STAT3 signaling axis.

### Depletion of SK1 impaired cargo sorting in FMC-derived EVs

**Fig. 6:**
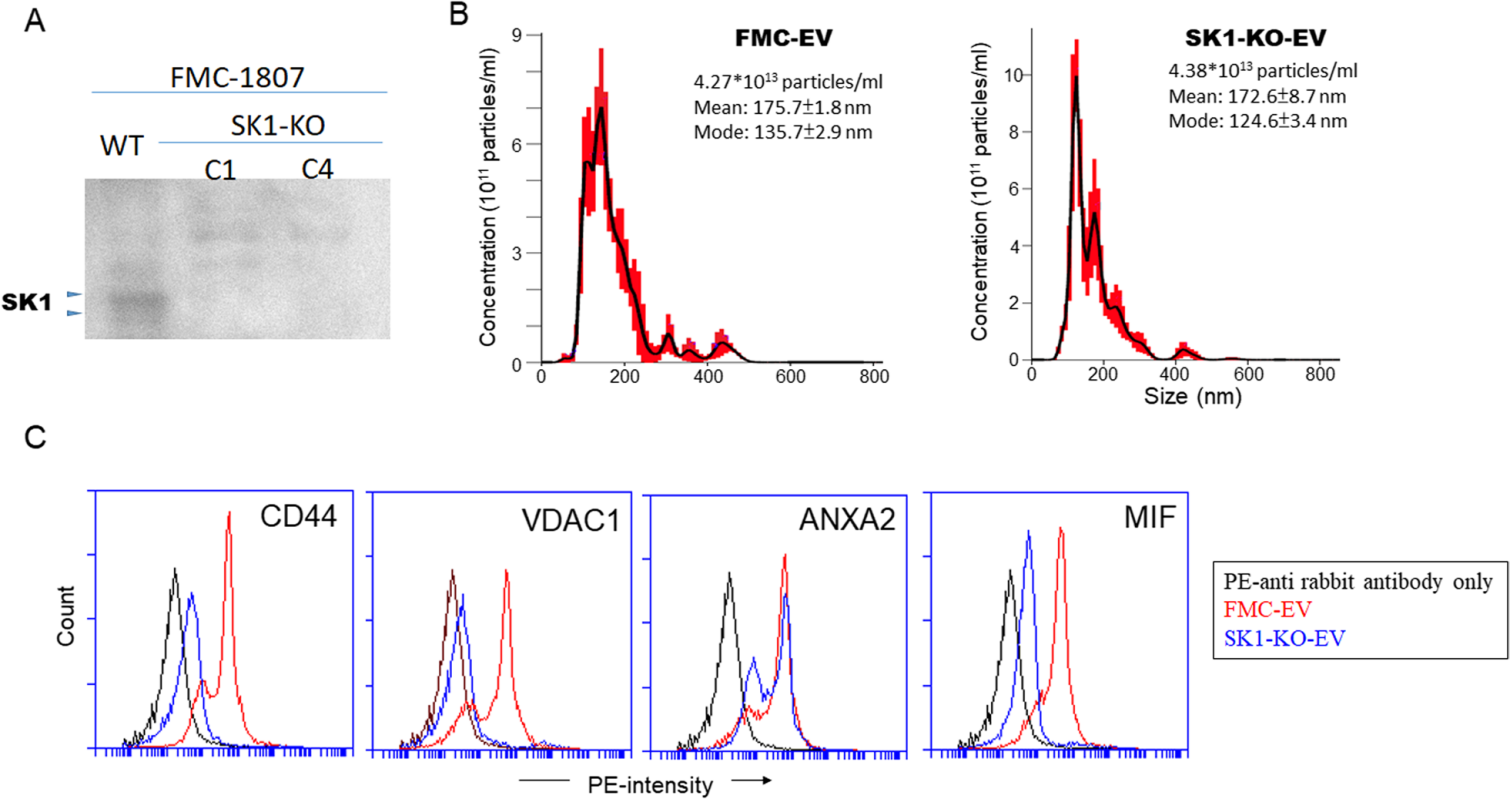
Depletion of SK1 impaired cargo sorting in FMC-EV. **A** Depletion of SK1 expression in two SK1-knockout (SK1-KO) clones (C1 and C4) derived from FMC-1807 was verified by Western blotting analysis. **B** The concentration and size distribution of SK1-KO EV (right panel) were comparable with that of FMC-EV (left panel). Representative data of nanoparticle tracking analysis are shown. **C** Semi-quantitation of EV content by flow cytometric analysis. Bead-coupled EV was stained with primary antibodies specific to CD44, VDAC1, ANXA2, or MIF, followed by incubation with appropriate PE-conjugated secondary antibodies

To better evidence the impact of SK1 on the pro-metastatic effect of FMC-EV, we employed a CRISPR/Cas9-based method to generate SK1-knockout (KO) FMC cells with FMC-1807. Western blotting analysis confirmed the deficiency of SK1 expression in two selected cell clones (Fig. 6A). Moreover, the mean diameter of EVs derived from SK1-KO cells was 172.6 ± 8.7 nm (Fig. 6B), indicating that depletion of SK1 had little effect on the size of FMC-EV. To evaluate whether SK1 conferred the pro-metastatic effect of FMC-EV via modulating the EV content, we conducted a proteomics analysis of protein components in FMC-EV (Additional file 1: Table S1) and selected four candidates which are reported as EV cargos and exert pro-metastasis functions, including the cluster of differentiation 44 (CD44) [24], voltage dependent anion channel 1 (VDAC1) [25], annexin A2 (ANXA2) [26], and macrophage migration inhibitory factor (MIF) [7, 27]. We coupled FMC-EV or SK1-KO EV with aldehyde/sulfate-latex beads and then labeled with specific antibodies followed by flow cytometric analysis. As shown in Fig. 6C, CD44, VDAC1, ANXA2, and MIF indeed were detected in FMC-EV. Notably, the amounts of CD44, VDAC1, and MIF were significantly reduced in SK1-KO EV. Data suggest that SK1 can modulate the cargo sorting in FMC-EV where the pro-metastatic proteins are delivered and contribute to FMC metastasis.

### SK1-KO EV failed to induce activation of HSC and the hepatic metastasis of FMC

**Fig. 7:**
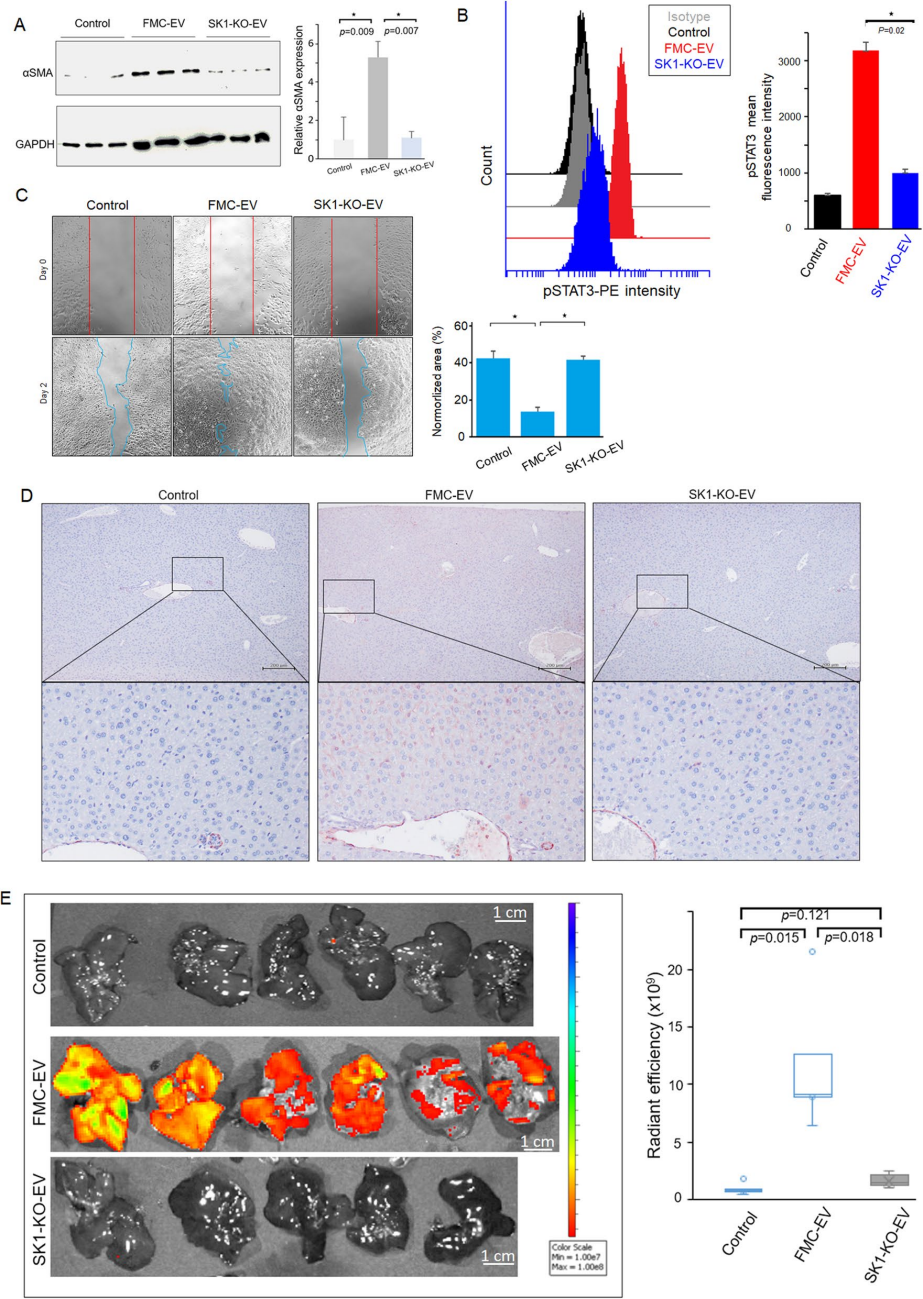
SK1-KO EV failed to activate HSC and promote FMC metastasis to liver. **A** SK1-KO EV failed to activate HSC. LX-2 cells were incubated with PBS (control), FMC-EV, or SK1-KO EV for 24 h. The expression of α-SMA in LX-2 cells was analyzed by Western blotting. The samples were analyzed in triplicate. Protein band intensity was quantified using ImageJ (right panel) **B** SK1-KO EV failed to activate STAT3 in HSC. Flow cytometric analysis of the p-STAT3 level in LX-2 cells after treatment with PBS (control), FMC-EV, or SK1-KO EV. The mean fluorescence intensity of p-STAT3 is shown (right panel). *n* = 3 in each group. **C** SK1-KO EV failed to promote migration of HSC. LX-2 cells incubated with PBS (control), FMC-EV, or SK1-KO EV were grown in the Culture-Insert (ibidi) for a wound healing assay. Images were acquired after 48 h, and the closure area of the wound was quantified using ImageJ. *n* = 3 in each group. **D** Immunohistochemical analysis of α-SMA (red) in livers taken from NPG mice intraperitoneally injected with PBS (control), FMC-EV, or SK1-KO EV for three weeks. The liver sections were counterstained with hematoxylin (blue). **E** Representative results of ex vivo imaging of the livers harvested from the mice intraperitoneally injected with PBS (control), FMC-EV, or SK1-KO EV, followed by intrasplenic injection with FMC-1807-RFP. The fluorescence intensity of FMC-1807-RFP in liver was acquired as radiant efficiency. *n* = 6 in each group. Quantitative data are presented as the mean ± SD. The Student’s t-test was used to determine the statistical significance

To confirm the impact of SK1 on the EV-induced activation of HSC, we first incubated LX-2 cells with FMC-EV or SK1-KO-EV, and examined the induction of α-SMA expression in LX-2 cells. As shown in Fig. 7A, SK1-KO EV was unable to induce α-SMA expression in LX-2 cells compared with those treated with FMC-EV. Moreover, flow cytometric analysis showed that SK1-KO EV failed to activate STAT3 in LX-2 cells (Fig. 7B), confirming that FMC-EV induced STAT3 activation in recipient LX-2 cells via the EV-delivered SK1. We further conducted a wound healing assay to examine the migration ability of LX-2 cells incubated with FMC-EV. As shown in Fig. 7C, FMC-EV promoted the migration of LX-2 cells compared with the PBS-treated control. In contrast, SK1-KO EV showed significantly impaired effect on LX-2 migration. Results collectively demonstrate that SK1 is essential for the activation of HSC by FMC-derived EVs.

We next verify the above SK1-mediated effects in vivo by intraperitoneally injecting mice with FMC-EV, SK1-KO EV, or PBS for three weeks and subsequently analyzed the expression of α-SMA in liver by immunohistochemistry. As shown in Fig. 7D, α-SMA expression was enhanced in liver preconditioned with FMC-EV compared with the PBS-treated control. However, α-SMA expression remained unchanged in liver preconditioned with SK1-KO EV, implicating that the EV-delivered SK1 instigated HSC activation. Furthermore, we investigated the impact of EV-delivered SK1 on hepatic PMN formation by detecting hepatic colonization of FMC-1807-RFP intrasplenically injected into the mice preconditioned with FMC-EV or SK1-KO EV. As shown in Fig. 7E and Additional file 1: Figure S2, FMC-1807-RFP significantly colonized in liver preconditioned with FMC-EV compared with the PBS-treated controls, in line with the previous results. In significant contrast, FMC-1807-RFP failed to colonize in liver preconditioned with SK-KO EV. Together, our data evidence the essential role of SK1 delivered by FMC-EV in structuring a hepatic PMN through activating HSC.

## Discussion

While circulating EV has been considered as a potential biomarker for FMC [28, 29], the correlation between FMC-derived EV and FMC metastasis has yet been characterized. In this study, we unveiled the liver tropism of FMC-EV which confers hepatic metastasis of FMC through activating HSC to prime a PMN. HSC is a multifunctional cell type which secretes ECM components in the liver upon activation by inflammatory and pro-fibrotic cytokines and consequentially transforms into myofibroblast [4, 21, 22]. Notably, active HSC has been implicated in contributing to liver metastasis of cancer by remodeling the hepatic ECM to support disseminated cancer cell colonization and proliferation, and eventually switching cancer dormancy [4, 26–28]. On the other hand, it has been shown that endothelial cells could regulate the phenotype of HSC via endothelial cell-derived exosomes containing SK1 [30]. Moreover, deletion of SK1 in fibroblasts abrogates fibroblast activation into myofibroblast and secretion of ECM proteins, indicating that the SK1-mediated signaling is pivotal for the myo-fibroblastic conversion and tumor-promoting effects of cancer-associated fibroblasts [24, 25].

We previously demonstrated that SK1 is highly expressed in FMC, and high levels of SK1 correlated with poor prognosis of FMC [24], in line with the notion that overexpression of SK1 in human triple-negative breast cancer is associated with an unfavorable disease outcome [19]. Furthermore, SK1 has been shown to promote carcinoma metastasis in multiple mouse models [20–22]. This effect likely attributes to the release of SK1 into the extracellular medium where SK1 catalyzes the biosynthesis of S1P, a proangiogenic signaling molecule. SK1 is a leaderless protein which can be secreted to the tumor microenvironment via an unconventional mechanism, i.e. EV. SK1 is shown to be present in a catalytically active form in vesicles shed by human hepatocellular carcinoma cells and human breast carcinoma cells [31]. Consequently, the vesicle-packaged SK1 can convert the membrane sphingosine to S1P which is released in the extracellular space and its level correlates with disease progression. For instance, the S1P level in the human fibrotic liver increases through upregulation of SK1 expression, and blood S1P levels are significantly associated with the stage of liver fibrosis in chronic liver diseases [32, 33]. More importantly, it has recently been demonstrated that ovarian cancer-derived EVs can drive T cell exhaustion in the tumor microenvironment through SK1/S1P-mediated signaling and impacting immunotherapy outcomes [34]. Although S1P was undetectable in FMC-EV in this study, it is plausible that FMC-EV-delivered SK1 catalyzes the sphingosine in the target HSC after HSC ingests the EV. It is worthy to elucidate the mechanism underlying the uptake of FMC-EV-packaged SK1 in HSC and transduction of the SK1-mediated signaling driving HSC activation.

We also showed that SK1 could play an important role in protein cargo sorting of FMC-derived EV by conducting proteomics analyses of the EV contents. We validated that CD44, VDAC1, and MIF were significantly reduced in SK1-KO EV. Data suggest that SK1 could modulate the sorting of protein cargos into FMC-EV. It has been shown that exosomal formation inside multivesicular bodies (MVBs) depends on S1P [35, 36]. Specifically, the S1PR1 is present on MVBs and its sustained activation controls exosomal maturation and cargo sorting. Moreover, downregulation of either S1PR1 or the S1P synthesizing enzyme, SK2, decreases CD63, CD81, and flotilin-2 content in exosomes [35, 36]. We assume that SK1 could function in a similar way for the cargo sorting of EV derived from FMC. On the other hand, we observed that FMC cells and primary FMC in the xenograft mouse model were also susceptible for uptake of FMC-EV (Fig. 3C). While FMC-EV appeared to have effect on the tumor load of receiving primary FMC, the pro-metastatic protein cargos delivered by FMC-EV might promote primary FMC cells to metastasize to the liver where HSC had been primed by FMC-EV to restructure a hepatic PMN.

This study does possess limitations. Firstly, the reliance on a FMC xenograft model utilizing immunocompromised mice may not accurately represent the intricate interactions within the tumor microenvironment, especially concerning interactions with the host's immune system. The potential role of FMC-EV in immune modulation or evasion, which could be pivotal in metastasis, remains unexplored. Secondly, while we identified alterations in the levels of several proteins, such as CD44, VDAC1, and MIF, in the SK1-KO EV, the precise functional significance and contribution of these proteins to the pro-metastatic effects of FMC-EV have yet to be determined.

## Conclusions

This study reveals for the first time that SK1-expressing FMC-EV can prime HSC activation to establish a hepatic PMN and thereby accelerates hepatic metastasis of FMC. The results provide a new therapeutic route to inhibit FMC metastasis to liver by targeting of EV-delivered SK1.

## Methods

### Culturing of FMC cell lines

Two FMC cell lines, FMC-1807 and FMC-1806, were respectively established from two FMC patient-derived xenografts (PDX) [37]. The human breast cancer cell (MDA-MB-231), mouse mammary epithelial cell (NMuMG), mouse hepatocytes (FL83B and BNL CL.2), mouse macrophage (RAW 264.7), mouse endothelial cell (SVEC4-10), and human HSC (LX-2) were purchased from Bioresource collection and research center (Taiwan). FMC-1807-RFP, FMC-1806-RFP, or MDA-MB-231-RFP stable lines were generated by infecting the cells with a VSVg pseudotyped lentivirus delivering a gene encoding red fluorescence protein (RFP) purchased from the National RNAi Core Facility, Academia Sinica (Taipei, Taiwan). All cell lines were cultured in Dulbecco’s modified Eagle’s medium (DMEM; Gibco, Waltham, MA, USA) supplemented with 10% fetal bovine serum (FBS; HyClone Laboratories, Logan, USA) at 37°C with 5% CO_2_. FL83B cells were cultured in Ham's F12K medium (Gibco, NY, USA) plus 10% FBS.

### Generation of SK1-KO FMC cell lines

Two guide RNAs (gRNAs) targeting feline SK1 were designed by using the Guide De-sign Resources (http://crispr.mit.edu/) with the given score of 75. The forward gRNA sequence is 5′- GACGATGAGGCACAGCCCCG-3′, and the reverse gRNA sequence is 5′- GTGGCCCTTCCGCCCTTGCC. The gRNA oligonucleotides were cloned into the pAll-Cas9 D10A.Ppuro vector. The resulting gRNA-expressing vector was assembled in a VSVg pseudo-typed lentivirus which was generated by the National RNAi Core Facility, Academia Sinica Taiwan. For generation of SK1-KO FMC cells, FMC-1807 cells were infected with the above lentivirus in the presence of 8 μg/ml polybrene (Sigma-Aldrich, H9268, Osaka, Japan). At 16 h post-infection, cells were selected with 2 μg/ml puromycin (Santa Cruz Biotechnology, Dallas, TX, USA) for two weeks. Live cells were serially diluted and seeded into a 96-well culture plate. Single-cell clone grown was cultivated in the presence of 1 μg/mL puromycin until a stable cell clone (SK1-KO FMC-1807) was obtained. The null expression of SK1 was verified by Western blotting and flow cytometry analyses.

### Isolation of EVs

EV-depleted FBS was prepared by centrifugation at 100,000 g for 90 min at 4°C using Beckman Optima L-70 ultracentrifuge with a SW-28 Ti rotor and collection of the resultant supernatant. FMC-1807 cells or MDA-MB-231 cells were grown to 70% confluency in a 100 mm culture dish, and then the culture medium was replaced by DMEM supplemented with 10% EV-depleted FBS after wash with 1 × PBS. The conditioned medium (CM) was collected two days later and centrifuged at 800 g for 10 min, followed by centrifugation at 2,000 g for 30 min to remove cellular debris. The EVs were further isolated by using a size-exclusion chromatography method. Briefly, the qEV column (Izon Science, Christchurch, New Zealand) was rinsed with PBS, and the EV-enriched samples were placed on the top of the column for fractionation. All fractions (fraction 1–12) were collected and the EV-rich fractions were determined by Western blotting analysis of EV markers, CD9 and CD63.

### Scanning electron microscopy (SEM)

The EV sample was fixed by 2% EMS-quality paraformaldehyde and added onto a cleaned silicon chip. The sample was subsequently mounted on a SEM stage with carbon paste, follow by coating with a gold–palladium alloy. The grids were observed using a field emission-SEM microscope (Ultra Plus, Zeiss).

### Nanoparticle tracking analysis

Size distribution and concentration of EVs were measured by Center for Micro/Nano Science and Technology (National Cheng Kung University, Taiwan) using a Nanosight Lm10 Hs microscope (Malvern Panalytical, UK).

### Immunohistochemistry

IHC was performed using the following protocols. In brief, antigens in paraffin-embedded tissue sections were retrieved with sodium citrate buffer (10 mM sodium citrate, pH 6.0, 0.05% Tween 20), and incubated for 1 h at room temperature with the primary antibodies as follows: anti-vimentin (clones V9; Cell Signaling Technology, Danvers, MA, USA; 1:100 dilution), anti-cytokeratin 5/6 (clone D5/16B4; Thermo Fisher Scientific, Carpeteria, CA, USA; 1:100 dilution), or anti-α-SMA (clone EPR5368; ABclonal, Boston, MA, USA; 1:500 dilution). The tissue sections were subsequently stained with DAB (Vector Laboratories, Burlingame, CA, USA) and Gill’s hematoxylin.

### Western blot analysis

Cell or EV lysates were extracted in ice-cold lysis buffer (20 mM Tris–Cl, 137 mM NaCl, 2 mM NaPPi, 25 mM β-glycerophosphate, 1 mM Na_3_VO_4_, 10% glycerol, 10 μM leupeptin, 2 mM EDTA, 0.77 μM aprotinin, 10 mM PMSF, 0.5 mM DTT, and 2 mM benzamidine; pH 7.4). Protein concentration of the above samples was measured by using the Pierce BCA Protein Assay Kit (Thermo Scientific, Rockford, IL, USA). Protein samples were loaded and separated on 8–10% polyacrylamide SDS gels, and transferred to Immuno-blot PVDF membranes. The PVDF membranes were blocked with 5% skim milk for 1 h at room temperature and incubated with primary antibodies overnight at 4°C. The antibodies used in this study include anti-CD63 (clone H5C6; BioLegend, San Diego, CA, USA), anti-CD9 (clone HI9a; BioLegend, San Diego, CA, USA), anti-fibronectin (A12932, ABclonal, Woburn, MA, USA), anti-SK1 (clone D1H1L; Cell Signaling Technology, Beverly, MA, USA), anti- S1P (ab224618; Abcam, Cambridge, MA, USA), anti-α-SMA (clone EPR5368; Abcam, Cambridge, UK), and anti-GAPDH (clone 14C10; Cell Signaling Technology, Danvers, MA, USA). The membranes were washed with Tris-buffered saline containing 0.05% Tween 20 and incubated with appropriate secondary antibodies for 1 h at room temperature. Chemiluminescence signals of immunoblots were developed with ECL substrate (Merck Millipore, Burlington, MA, USA) and detected using ImageQuant LAS 4000 (GE Heathcare, Sweden). Intensity of the bands was normalized to GAPDH and was quantified using ImageJ software 1.52a.

### Assay for cellular uptake of EVs

Cells (1 × 10^4^) were incubated with CD63-GFP-expressing EVs (1 × 10^8^ particles) for indicated time and subsequently applied for flow cytometry analysis (BD Accuri C6 Plus). The percentage of GFP-positive cells which ingested the CD63-GFP-expressing EVs was calculated according to the flow cytometry data. For microscopic imaging of EV uptake, cells (1 × 10^5^) were incubated with CD63-GFP-expressing EVs for indicated time. The uptake of EVs inside cells were observed using the EVOS M5000 Imaging System (Thermo Fisher Scientific).

### Flow cytometry analysis

Cells were resuspended in ice-cold PBS buffer containing 0.5% BSA, fixed and then permeabilized for intracellular staining using phycoerythrin (PE)-conjugated anti-phosphorylated STAT3 at Tyr705 (13A3-1, BioLegend, San Diego, CA, USA) or anti-α-SMA (1A4, BioLegend, San Diego, CA, USA) on ice for 30 min containing with PhosStop phosphatase inhibitor (Roche, Madison, WI, USA).

For quantitation of the EV contents, EV samples were incubated with aldehyde/sulfate-latex beads (Invitrogen, Carlsbad, CA, USA) at room temperature for 30 min. Bead-coupled EVs were pelleted by centrifugation at 2,000 g for 10 min, resuspended in PBS and subsequently stained with primary antibodies: anti-CD44 (clone IM7, Merck Millipore, Rahway, NJ, USA), anti-VDAC1 (clone N152B/23, BioLegend, California, USA), anti-ANXA2 (clone W18057A, BioLegend) or anti-MIF (clone 10C3, BioLegend, San Diego, CA, USA) for 30 min and washed with PBS twice. The bead-coupled EVs were then incubated with appropriate fluorescent dye-conjugated secondary antibodies (P-2771MP and M30004-1, Thermo Fisher Scientific, Waltham, MA, USA) for 30 min and washed twice.

The staining of samples was analyzed using the Accuri C6 plus flow cytometer and quantified using the CSampler plus software (BD Biosciences, USA). The sample without primary antibody incubation was used as a negative control.

#### Assay of HSC activation

LX-2 cells were starved in serum-free DMEM for two hours and then incubated with FMC-EVs (10^9^ particles) for 24 h in the presence or absence of SK1 inhibitors, 1 μM CAY10621 (13,371, Cayman, Ann Arbor, Michigan, USA) or 100 nM PF-543 (17,034, Cayman, Ann Arbor, MI, USA). The expression level of α-SMA (a profibrotic marker) in LX-2 cells was analyzed by Western blotting. For a cell proliferation assay, LX-2 cells treated as above were seeded into a 96-well plate (2,000 cells/well) and grown in the presence of the Cell Counting Kit-8 (Dojindo, Kumamoto, Japan) for two hours. The cell viability was measured by detecting the op-tical density of the reaction at 450 nm. For a wound healing assay, LX-2 cells were seeded into a Culture-Insert 2 Well (ibidi, Munich, Germany) placed in a 6-well culture plate for two days. Cell migration was assessed by manually measuring the remaining area of the wound using the ImageJ.

#### Statistical analyses

Results were expressed as means ± SD and analyzed for differences between two groups using two-tailed t-tests, assuming equal variances, with statistical significance set at p < 0.05. The statistical analyses were performed using IBM SPSS Statistics 20 software (IBM Corporation, USA).

#### Proteomics analysis of FMC-EV derived from parental or SK-depleted FMC cells

The cargo proteins in FMC-EV were identified by LC–MS/MS analysis using an UltiMate 3000 RSLCnano LC (Thermo Fisher Scientific) coupled with a TripleTOF® 6600 System (Applied Biosystems Sciex) at the Agricultural Biotechnology Laboratories of National Chung Hsing University. Purified EVs (10 μg) derived from cultured FMT-1807 cells were lysed in urea lysis buffer (8 M urea buffer in 50 mM ammonium bicarbonate) and then reduced with 4 mM Dithiothreitol and alkylated with 8 mM iodoacetamide). Protein samples were concentrated with a 10 kDa centrifugal spin filter (Sartorius, Göttingen, Germany) and digested with trypsin (enzyme/substrate ratio 1:100; Promega, Madison, WI, USA) at 37 °C overnight. The trypsin digestion was quenched with 5% formic acid, and the resulting peptides were cleaned-up on SepPak C18 cartridges (Waters Corporation, Milford, MA) and applied for proteome analysis. After trapping for 5 min in a flow rate of 0.05 ml/min in 100% solvent A (0.1% formic acid in H_2_O), peptides were eluted with a 160 min LC gradient from 10 to 36% solvent B (0.1% formic acid, 80% ACN) at a flow rate of 300 nL/min.

#### Database searching and bioinformatics

Tandem mass spectra were extracted and the charge state was deconvoluted and deisotoped by MASCOT Distiller software (Version 2.0; Matrix Science, Boston, MA, USA). All MS/MS samples were analyzed using MASCOT algorithm (version 2.1, Matrix Science against the NCBI-*Felis catus* and SwissProt animal database. The mass tolerances for parent and fragment ions were set as 0.03 Da and 0.05 Da, respectively. The enzyme was set as trypsin and up to one missed cleavage was allowed. Carbamidomethylation of Cys, oxidation of His, Trp, and Met, and deamidation of Asn and Gln were set as variable modifications during database search.

#### FMC xenograft-bearing mice and tissue collection

Animal care and all procedures were previously approved by the Institutional Animal Care and Use Committee (IACUC) of National Chung Hsing University (IACUC Number: 106–73^R3^ and 110–012). NOD/Prkdcscid/IL-2Rγnull (NPG) mice were purchased from BioLASCO (Taipei, Taiwan). Mice were housed in a 12-h light/dark cycle, with lights on from 7:00 am to 7:00 pm, and given ad libitum access to food and water. FMC tissue cubes (approximately 8 mm^3^) were surgically resected from an FMC-1807-PDX mice [23] and subsequently transplanted into the inguinal mammary fat pads of 8-week-old NPG mice under isoflurane anesthesia (induced into anesthesia with 4% isoflurane (Attane; Piramal Enterprises Limited, Telangan, India) for 2–5 min, then maintained by continuous inhalation of 1.5%-2% isoflurane). The mice were intraperitoneally injected with isolated FMC-EVs (10^10^ particles/100 µl of 1 × PBS, 6 mice) or blank 1 × PBS (6 mice) weekly since the third week post-transplantation. Tumor size was measured weekly using a caliper and calculated with the formula as length × (width)^2^ × ½. For collection of tissues, the mice were sacrificed by cervical dislocation under isoflurane anesthesia, and the lung, liver, spleen, and femur were collected and sectioned to examine FMC metastasis. For quantification of metastasized FMC, cross-sections of tissues collected from each mouse were stained with hematoxylin and eosin. Immunohistochemistry (IHC) of vimentin and cytokeratin 5/6 was used for determining the number and area of metastasized tumors. Histology and IHC images of each tissue specimen were acquired by using Aperio AT2 (Leica, USA) at 20 × magnification. Aperio ImageScope × 64 software was used to manually identify the tumor foci in tissue sections.

#### Biodistribution of CD63-GFP-expressing FMC-EVs in FMC xenograft-bearing mice

To generate an FMC cell line expressing EGFP-fused CD63, the cDNA encoding human CD63 fused with GFP purchased from Addgene (CD63-pEGFP C2, Addgene 62964) was subcloned into the pLAS2w.Ppuro lentiviral vector. The VSVg pseudotyped lentivirus was produced using HEK-293T cells which had been co-transfected with pCMVDR8.91, pMD.G, and pLAS2w.Ppuro-CD63-GFP. The virus production was performed at the National RNAi Core Facility, Academia Sinica Taiwan. FMC-1807 cells were infected with the above lentivirus in the presence of 8 μg/ml polybrene. At 16 h post-infection, cells were selected with 2 μg/ml puromycin for 4 weeks to obtain a stable cell line (CD63-GFP-FMC-1807) which were CD63-GFP-positive.

To trace the biodistribution of FMC-derived EVs in vivo, three FMC-1807 xenograft-bearing mice were injected intraperitoneally with CD63-GFP-expressing EVs (1 × 10^11^ particles/100 μl PBS) when tumors reached a volume of approximately 400 mm^3^. The primary tumor, lung, liver, spleen and kidney were harvested at 0, 12- or 24-h post-injection (3 mice were sacrificed at the indicated time point) for bioluminescent imaging. The imaging was performed using a Caliper IVIS SpectrumCT In Vivo Imaging System (Caliper Life Sciences, USA).

#### Colorization of FMC cells in an EV-conditioned pre-metastasis niche in the liver

NPG mice (six mice per group) were intraperitoneally injected with 1 × 10^10^ FMC-EVs, SK1-KO-EVs or PBS three times a week for three consecutive weeks. One mouse each group was sacrificed for detection of HSC activation or ECM remodeling in the liver. Under anesthesia with isoflurane, mice which had been shaved and disinfected were placed on a heating pad in a right-lateral recumbent position. A 1-cm incision was made in the upper-left abdominal wall to expose the spleen. The spleen was translocated onto a saline gauze dressing and gently injected with 1 × 10^5^ FMC-1807-RFP, FMC-1806-RFP, or MDA-MB-231-RFP cells in 50 μl of DMEM. The injection site was cauterized to curtail bleeding. The abdominal incision was closed with a 5–0 absorbable thread (EU-TEK, New Taipei, Taiwan). The mice were subcutaneously injected with 5 mg/kg Carprofen (Chunghwa Senior Care, Taipei, Taiwan) for analgesia and placed on a heating pad until recovered. After three weeks, the mice were sacrificed, and the livers were subjected to ex-vivo IVIS imaging to confirm FMC metastasis.

### Supplementary Information

**Additional file 1: Fig. S1**. Observation of CD63-GFP-expresing EV cellular uptake by FMC-1807 cell (upper) and LX-2 cell.** Fig. S2**. Representative results of ex vivo imaging of the lung, liver, kidney, spleen and heart harvested from the mice treated with PBS, FMC-EV, or SK1-KO EV, followed by intrasplenic injection with FMC-1807-RFP. **Table S1.** EV proteins from FMC-EV and SK1-KO EV.

## Data Availability

The data used during the current study are available from the corresponding author upon reasonable request.

## Abbreviations

*ANXA2*: Annexin A2
*α-SMA*: α-Smooth muscle actin
*CD44*: Cluster of differentiation 44
*ECM*: Extracellular matrix
*EV*: Extracellular vesicle
*FMC*: Feline mammary carcinoma
*HSC*: Hepatic stellate cells
*IL-6*: Interleukin-6
*MIF*: Macrophage migration inhibitory factor
*PMN*: Premetastatic niche
*S1P*: Sphingosine-1-phosphate
*SK1*: Sphingosine kinase 1
*STAT3*: Signal transducer and activator of transcription 3
*VDAC1*: Voltage dependent anion channel 1
