# Supplementary material for: Feline mammary carcinoma-derived extracellular vesicle promotes liver metastasis via sphingosine kinase-1-mediated premetastatic niche formation
Source: Lab Anim Res. 2023 Nov 9;39:27. doi: 10.1186/s42826-023-00180-5 (PMC10634095; doi:10.1186/s42826-023-00180-5)
Supplement: Supplementary file 1 — Additional file 1: Fig. S1. Observation of CD63-GFP-expresing EV cellular uptake by FMC-1807 cell (upper) and LX-2 cell. Fig. S2. Representative results of ex vivo imaging of the lung, liver, kidney, spleen and heart harvested from the mice treated with PBS, FMC-EV, or SK1-KO EV, followed by intrasplenic injection with FMC-1807-RFP. Table S1. EV proteins from FMC-EV and SK1-KO EV. [file 42826_2023_180_MOESM1_ESM.docx]

Supplemental figure 1


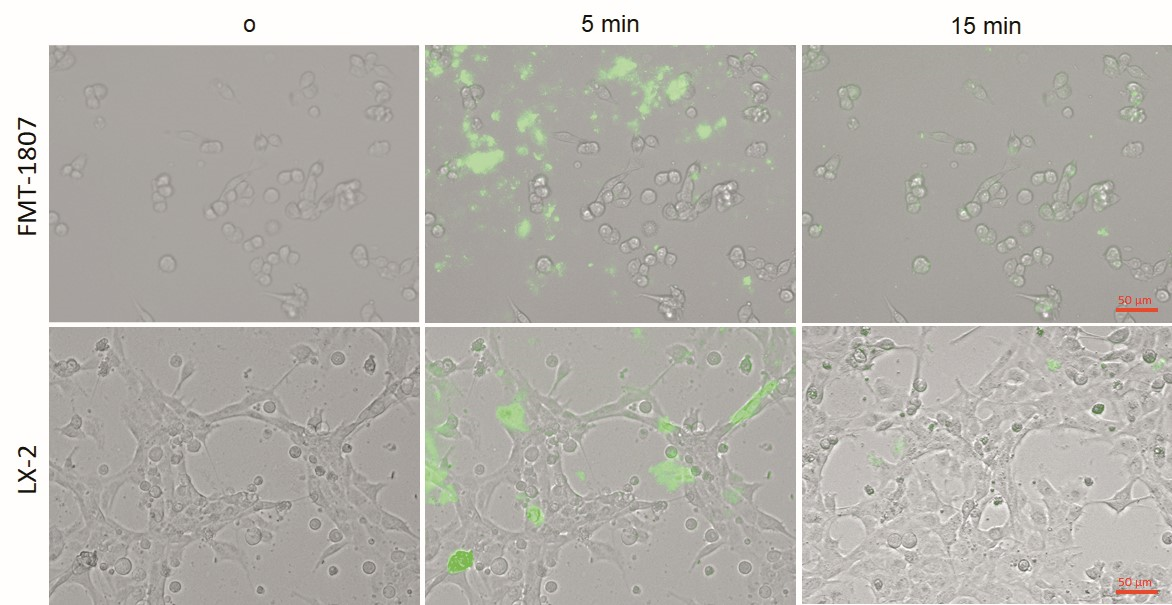


Observation of CD63-GFP-expresing EV cellular uptake by FMC-1807 cell (upper) and LX-2 cell (lower). Scale bar, 50 μm.

Supplemental figure 2


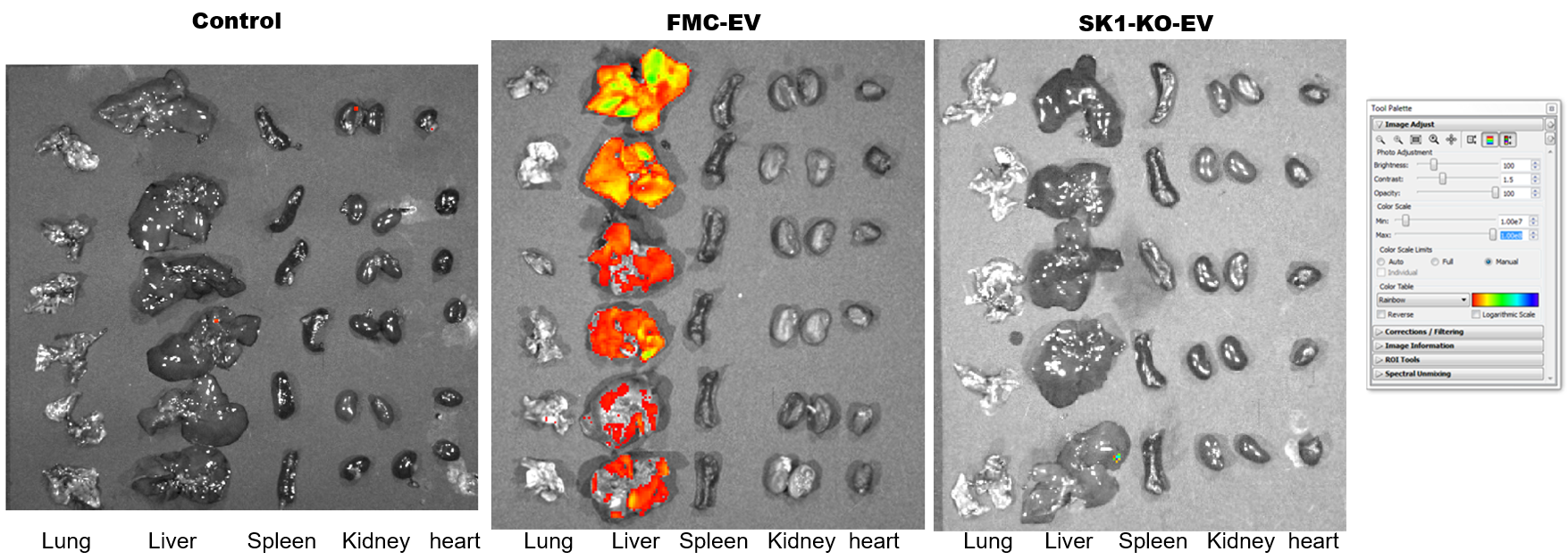


Representative results of *ex vivo* imaging of the lung, liver, kidney, spleen and heart harvested from the mice treated with PBS (control), FMC-EV, or SK1-KO EV, followed by intrasplenic injection with FMC-1807-RFP.

Supplementary Table 1: EV proteins from FMC-EV and SK1-KO EV, related to Figures 6.

| Accession |  | Score EV-FMC-1807 | Score EV-SK1-KO-FMC-1807 |
| --- | --- | --- | --- |
| A0A5K1VJR6 | HSP90AA1 | 2863.85 | 49.93 |
| A0A2I2U7X4 | HSP90AB1 | 2584.29 |  |
| A0A2I2UZM2 | HSPA8 | 2249.58 | 478.67 |
| M3W6I2 | ACTG1 | 1877.68 | 723.88 |
| M3VVM1 | EEF2 | 1399.09 |  |
| M3XC93 | ACTA1 | 1279.33 |  |
| M3X7Z9 | TUBB | 1060.77 |  |
| M3XF66 | EEF1A2 | 905.14 | 508.46 |
| M3XBC3 | EIF4A1 | 899.42 |  |
| M3W0Z9 | TUBB4B | 874.68 |  |
| A0A2I2U7Y0 | ALB | 719.58 | 511.79 |
| M3WXZ4 | HSPD1 | 706.91 |  |
| A0A2I2U9C6 | LDHB | 683.17 |  |
| M3WER4 | TUBA1A | 681.68 |  |
| A0A5F5XKD4 | CLTC | 627.02 | 803.84 |
| M3WK11 | HSP90B1 | 595.21 |  |
| M3WVC7 | YWHAZ | 577.24 |  |
| M3VW78 | LOC111558717 | 566.05 |  |
| M3WAH7 | DYNC1H1 | 554.85 |  |
| A0A5F5Y605 | TKT | 521.91 |  |
| M3X166 | PGK2 | 509.69 |  |
| A0A2I2UJH0 | PGK1 | 495.58 | 50.57 |
| A0A2I2U551 | AHCY | 489.93 | 125.56 |
| M3WJI8 | MSN | 472.97 |  |
| M3WFT4 | HSPA5 | 458.02 |  |
| A0A337RYI0 | PKM | 455.78 |  |
| A0A2I2V2A4 | TLN1 | 452.02 | 89.44 |
| A0A2I2U1D6 | HK1 | 412.39 |  |
| A0A337S8L1 | LDHA | 402.80 | 256.43 |
| M3X0G7 | FLNA | 396.00 |  |
| M3WZB0 | ATP5F1A | 395.09 |  |
| M3VZQ3 | TUFM | 371.32 |  |
| A0A337SSE8 | ALDOA | 368.16 |  |
| A0A5F5XDQ3 | ACTN1 | 360.12 |  |
| M3WAC5 | EEF1G | 328.36 |  |
| M3X6F7 | ACTN4 | 323.30 |  |
| M3VWK3 | UBA1 | 310.04 |  |
| M3W8I2 | YWHAQ | 293.26 |  |
| A0A2I2U1C5 | MYH9 | 279.88 | 51.85 |
| A0A337SIM6 | NME1 | 277.60 | 74.94 |
| M3VW05 | VCP | 274.60 | 818.23 |
| M3VXF8 | PABPC1 | 273.92 |  |
| A0A5F5XNI1 | TF | 266.92 | 208.05 |
| A0A2I2V266 | GSN | 243.31 | 49.66 |
| A0A337S8S6 | LOC101085453 | 241.16 | 230.41 |
| A0A5K1VP01 | LMNA | 223.71 |  |
| M3WND0 | PHB2 | 222.43 |  |
| M3WNF9 | CCT8 | 217.71 |  |
| A0A2I2UJB3 | RDX | 216.79 |  |
| M3WDN7 | EIF3A | 214.61 |  |
| A0A2I2U879 | LCP1 | 212.06 |  |
| A0A337SCS9 | YWHAE | 211.24 |  |
| A0A337SMT2 | ACLY | 206.51 |  |
| M3WFS1 | CCT3 | 205.22 |  |
| M3W8S0 | EZR | 204.90 |  |
| A0A5F5XZM5 | SLC25A3 | 202.53 |  |
| M3W668 | PDIA3 | 201.51 |  |
| A0A337S343 | CSE1L | 201.13 |  |
| A0A337SS16 | ENO1 | 199.40 | 127.89 |
| A0A2I2UQN3 | IQGAP1 | 194.13 |  |
| A0A337SG68 | HSPH1 | 192.84 |  |
| M3WIA8 | RPL27 | 191.73 |  |
| M3WFK0 | FASN | 188.58 | 89.48 |
| M3X9A1 | TPI1 | 187.84 |  |
| M3WXV4 | RAN | 186.51 | 170.02 |
| A0A0A0MPZ9 | PPIA | 181.80 | 139.22 |
| M3VZ17 | RPSA | 180.94 | 75.33 |
| M3WRD9 | RAB7A | 179.22 |  |
| A0A2I2U0T8 | P4HB | 176.78 |  |
| M3VZ89 | TCP1 | 176.46 |  |
| A0A5F5XGE0 | ATP1A1 | 175.16 |  |
| A0A5F5XP79 | HNRNPU | 170.43 |  |
| A0A2I2V2Q9 | LOC101087230 | 166.00 |  |
| A0A337SH94 | GANAB | 164.42 |  |
| M3WVY3 | NCL | 163.45 |  |
| A0A337SEF0 | CFL2 | 159.56 |  |
| A0A337S3P6 | NPM1 | 155.50 |  |
| A0A2I2UXU4 | VDAC1 | 155.08 |  |
| M3W9K0 | PYGB | 152.83 |  |
| A0A337RTX3 | PCBP2 | 152.81 |  |
| M3W2B4 | VARS1 | 152.46 |  |
| Q6BDL8 |  | 149.65 |  |
| M3WJC5 | CCT7 | 149.23 |  |
| A0A2I2UI32 | HNRNPK | 147.61 |  |
| M3W345 | HKDC1 | 145.82 |  |
| A0A2I2UE49 | UBC | 145.27 | 47.06 |
| Q0QEN3 | ATP5B | 144.27 |  |
| A0A337S4V3 | GAPDH | 142.90 |  |
| M3WFE1 | SSB | 141.09 |  |
| M3X7H4 | LOC101100947 | 140.01 |  |
| A0A337SEV4 | KHSRP | 138.32 |  |
| M3XBV5 | CANX | 137.00 |  |
| Q0QEK8 | CS | 135.66 |  |
| M3VW27 | RPS25 | 132.49 |  |
| M3W0R7 | RANGAP1 | 131.14 |  |
| M3VWG1 | YWHAG | 131.13 |  |
| M3WLL8 | SERPINC1 | 128.78 | 106.09 |
| M3WJ18 | MCM2 | 125.95 |  |
| A0A2I2U7C7 | SHMT2 | 125.33 |  |
| M3WXK0 | KPNB1 | 124.95 |  |
| M3X2J1 | VDAC2 | 124.13 |  |
| A0A337SBY1 | CNN2 | 123.94 |  |
| M3WRT2 | RPL8 | 123.27 |  |
| A0A337STF2 | PARK7 | 122.65 | 45.52 |
| M3WHB4 | GDI2 | 120.51 |  |
| M3W8D9 | PSMD2 | 117.52 | 294.77 |
| M3X557 | AFP | 116.54 |  |
| M3WEL8 | PFN1 | 113.94 |  |
| A0A2I2U004 | CLUH | 113.16 |  |
| A0A2I2UGY2 | RPL24 | 112.61 |  |
| A0A337S3P4 | DDX5 | 112.26 |  |
| A0A2I2V252 | ST13 | 109.71 |  |
| A0A2I2UU11 | CD44 | 108.96 |  |
| M3W9X1 | PDIA4 | 108.93 |  |
| Q18PA2 | LRP | 108.34 |  |
| A0A337S2E2 | RPL7 | 108.04 |  |
| M3W1I3 | MIF | 107.85 |  |
| A0A2I2UBH3 | PPIB | 107.64 |  |
| M3W5T1 | DLST | 104.14 |  |
| M3VWJ8 | VPS35 | 103.10 |  |
| A0A2I2UY85 | TPM3 | 102.76 |  |
| M3WF04 | COPB1 | 101.80 |  |
| A0A2I2UZ67 |  | 99.51 |  |
| M3VYZ8 | XPO1 | 98.82 | 82.18 |
| A0A2I2U188 | CAD | 97.81 |  |
| A0A2I2UWE4 | NUP93 | 95.74 |  |
| A0A2I2UFN2 | LOC101084134 | 95.68 |  |
| M3WP87 | ACO2 | 94.31 |  |
| A0A337SK71 | RPLP0 | 94.12 |  |
| A0A5F5XZJ0 | ARHGEF15 | 93.77 |  |
| M3WGN8 | RPL4 | 93.38 |  |
| A0A5F5XQH7 | RBBP4 | 92.91 |  |
| A0A2I2U8V4 |  | 91.79 |  |
| M3X983 | PSMA3 | 90.77 |  |
| M3W4R9 | RPL3 | 90.65 |  |
| M3X3I1 | PSAT1 | 89.94 |  |
| M3W501 | STIP1 | 89.69 |  |
| A0A5F5XMX0 |  | 89.24 |  |
| A0A337S231 | NASP | 88.35 |  |
| M3W3E7 | A2M | 88.33 | 43.62 |
| M3W114 | PLP2 | 88.25 |  |
| A0A5F5XWI1 | TOMM70 | 88.16 |  |
| A0A337SLC1 | GCN1 | 87.79 |  |
| M3VWM9 | FAU | 87.75 |  |
| A0A5F5Y328 | A2M | 86.20 | 56.62 |
| A0A5F5XE80 | EIF3E | 86.15 |  |
| A0A337S8K2 | PCMT1 | 86.05 |  |
| A0A2I2U093 | RPL5 | 85.88 |  |
| A0A337SMD7 | PSMD13 | 85.80 |  |
| A0A337SDC1 | HNRNPH1 | 85.45 |  |
| A0A2I2UW01 | CCT5 | 85.04 |  |
| A0A2I2U6J8 | LOC101091307 | 84.82 | 700.23 |
| A0A5F5XUS8 | TTLL12 | 83.95 |  |
| A0A337SJC3 | G6PD | 83.64 |  |
| A0A2I2U4W2 | RPL7A | 82.94 |  |
| A0A337RU36 | SET | 81.99 |  |
| M3WYU5 | EIF2S1 | 81.75 |  |
| Q6BDL3 |  | 81.22 |  |
| A0A337SJM7 | SFPQ | 80.52 |  |
| A0A337SID0 | SRSF2 | 80.28 |  |
| A0A2I2U0U0 | SRSF6 | 80.16 |  |
| A0A5F5XQX5 | TAGLN2 | 79.69 |  |
| M3VXX1 | TPT1 | 79.56 |  |
| A0A5F5XKM9 | HIST1H1E | 79.37 |  |
| M3W1E8 | RPL23A | 78.84 |  |
| A0A337S5C0 | ANXA5 | 78.47 |  |
| M3VZG0 | SUMO2 | 78.10 |  |
| A0A337S4H3 | GOT2 | 78.09 |  |
| A0A2I2UKJ6 | CHCHD3 | 77.53 |  |
| A0A5F5XQD3 | MTHFD1 | 76.98 |  |
| A0A337SHK2 | FETUB | 76.81 |  |
| M3WLD5 | NONO | 76.23 |  |
| M3W3Z6 | EIF3B | 75.75 |  |
| A0A337SEY0 | MDH1 | 74.88 |  |
| A0A5F5XU66 | DDX17 | 74.83 |  |
| A0A2I2UNG3 | RPL22 | 73.64 |  |
| M3WG24 | SERPINH1 | 73.48 |  |
| A0A2I2UQU1 | CBR3 | 72.80 |  |
| M3WAQ4 | ATAD3A | 72.77 |  |
| A0A5F5XER4 | SND1 | 72.15 |  |
| A0A2I2UXL1 | RPS3 | 71.62 |  |
| M3WA40 | LARS1 | 71.61 |  |
| A0A337RZ59 | EIF5A | 71.53 |  |
| A0A337S990 | H2AFV | 71.44 | 49.50 |
| A0A337SQZ3 | EEF1D | 70.96 |  |
| A0A5F5XSU7 | FXR1 | 70.06 |  |
| A0A337SSW0 | AIFM1 | 69.34 |  |
| M3W5Z5 | EIF2S3 | 69.33 |  |
| A0A337SNJ1 | PSMA6 | 68.78 | 158.45 |
| A0A5F5XG76 | ATP5PO | 68.73 |  |
| Q7YS39 |  | 68.73 |  |
| A0A5F5Y566 | ATIC | 68.36 |  |
| M3WUF3 | SYNCRIP | 68.22 |  |
| A0A5F5XGI7 | HSPA4 | 68.05 | 59.39 |
| A0A2D0UF13 | PSMB4 | 67.99 |  |
| A0A337S3U6 | UGDH | 67.41 |  |
| M3WB84 | GART | 67.40 |  |
| A0A337SJB5 | PHGDH | 67.21 | 62.13 |
| A0A2I2V3P6 | DPYSL2 | 67.17 |  |
| M3X5Z4 | NPEPPS | 66.96 |  |
| A0A5F5XXH1 | HSPA9 | 66.91 |  |
| A8WA76 | PHB | 66.66 |  |
| A0A2I2UDQ0 | GFPT1 | 65.77 |  |
| M3XCB8 | STOM | 65.68 | 195.50 |
| A0A5F5XSE6 | RPL35 | 65.32 |  |
| A0A337SR88 | CAPZB | 65.19 |  |
| A0A2I2U8P7 | H2BC21 | 64.91 | 101.20 |
| M3W8G3 | CTSD | 64.61 |  |
| M3XA07 | PSMD11 | 64.17 | 137.97 |
| M3WD17 | RPL12 | 64.00 |  |
| A0A2I2U5I7 |  | 63.93 |  |
| M3VVG6 | IARS | 63.79 |  |
| A0A2I2UEP1 | WDR1 | 63.77 |  |
| A0A5F5XK17 | HSPB1 | 63.64 |  |
| M3WVD0 | RAB11A | 63.52 |  |
| M3XAB2 | YBX1 | 62.65 |  |
| M3WNY6 | CLIC1 | 62.32 |  |
| M3W4B8 | PSME2 | 62.25 |  |
| M3WAY9 | RPL13A | 62.16 |  |
| M3XCV3 | VCL | 61.60 |  |
| A0A5F5XBT2 | EPRS1 | 61.46 |  |
| A0A2I2U374 | SEPTIN11 | 61.37 |  |
| A0A337S0U0 | HADHB | 61.35 |  |
| A0A5F5XK45 | DMRTC2 | 61.34 |  |
| A0A337SSM4 | RAB5B | 61.10 |  |
| A0A5F5XFT8 | LRPPRC | 61.07 |  |
| A0A2I2UDM1 | PSME3 | 61.00 |  |
| A0A337RWV9 | DLD | 60.82 |  |
| M3WP20 | PPM1G | 60.67 |  |
| A0A384DV38 | RPL17 | 60.62 |  |
| A0A337SRW5 | CDC42 | 60.61 |  |
| M3VXW7 | UQCRC1 | 60.45 |  |
| A0A2I2UFE0 | DSTN | 60.21 |  |
| A0A337SEG2 | FLNB | 59.44 |  |
| M3W7V0 | RPL18A | 59.41 |  |
| A0A337SB80 | RACK1 | 59.29 |  |
| A0A2I2UIN5 | DNAJC13 | 59.29 |  |
| A0A337SXE8 | ARL8A | 59.01 |  |
| M3VWP9 | COPB2 | 58.87 |  |
| A0A2I2UTM3 | PARP1 | 58.81 |  |
| M3VYD8 | ARPC2 | 58.67 |  |
| M3WNP8 | RENBP | 58.52 |  |
| M3WN96 | SPNS1 | 58.43 |  |
| A0A2I2UB94 | MAP2K2 | 57.44 |  |
| M3VY98 | GSS | 56.93 |  |
| A0A337S9C0 | SLC25A10 | 56.73 |  |
| A0A5F5Y1Z7 | PPFIBP1 | 56.60 |  |
| A0A337SU54 | HARS2 | 56.57 |  |
| A0A337SPM1 | RPS9 | 56.03 |  |
| A0A5F5Y0A1 |  | 55.85 |  |
| A0A337SGK2 | DHX9 | 55.71 |  |
| A0A5F5Y6C4 | HNRNPDL | 55.48 |  |
| M3WDU7 | RPS16 | 55.26 |  |
| M3WN94 | SGTA | 55.13 |  |
| A0A2I2UJ17 | MATR3 | 55.08 |  |
| M3WNX8 | XRCC5 | 54.87 |  |
| A0A337S256 | QDPR | 54.79 |  |
| M3X4J3 | RAP1A | 54.74 |  |
| M3W1X0 | USP5 | 54.51 |  |
| A0A5F5XDR4 | IPO5 | 54.40 | 79.56 |
| A0A337RU72 | NUP155 | 54.39 |  |
| M3WDA8 | HDLBP | 54.10 |  |
| M3W454 | DENND6A | 54.04 |  |
| A0A2I2UIG2 |  | 54.00 | 51.68 |
| A0A2I2UMT1 | DDX39A | 53.81 |  |
| M3WDL6 | RPS15A | 53.66 |  |
| A0A337RWC2 | ILF2 | 52.95 |  |
| M3WG15 | SF3A1 | 52.78 |  |
| M3WSH0 | SNRPB2 | 52.47 |  |
| A0A5F5XSJ8 | IMMT | 52.46 |  |
| A0A2I2U399 | DNAJA1 | 52.39 |  |
| M3WLY0 | STT3A | 52.32 |  |
| A0A5F5XG38 | RPS6 | 52.16 | 49.33 |
| M3WJL5 | SUPT16H | 52.08 |  |
| M3WAC7 | MTA2 | 51.93 |  |
| Q6B831 | H4C9 | 51.89 |  |
| M3W8B7 | UQCRC2 | 51.48 |  |
| A0A337SS24 | CSDE1 | 51.46 |  |
| A0A337SPI3 | ARF3 | 51.42 |  |
| A0A337S862 | HYOU1 | 51.41 |  |
| A0A5F5Y306 | SEC61A2 | 51.29 |  |
| M3XFB8 |  | 51.13 |  |
| A0A2I2UQT2 |  | 50.79 |  |
| M3WSA0 | HRAS | 49.99 |  |
| A0A5F5XGT7 | CAPN2 | 49.83 |  |
| M3X8E4 | ERP29 | 49.74 |  |
| A0A337SWX8 | CCT2 | 49.71 |  |
| A0A337S8V1 | PSMD5 | 49.62 | 52.89 |
| M3WK60 | CAPNS1 | 49.61 |  |
| M3XET1 | PSMA8 | 49.56 |  |
| A0A337SF71 | RPS2 | 49.53 |  |
| M3XE20 | RPS8 | 49.43 |  |
| A0A2I2UHR0 | MYL6 | 49.35 |  |
| A0A0A0MQ02 | LOC101097290 | 49.06 | 295.18 |
| M3X5Q2 | FUS | 48.98 |  |
| M3WX07 | AK1 | 48.70 |  |
| A0A337RZ29 | ALDH18A1 | 48.63 |  |
| A0A5F5Y6U8 | EEA1 | 48.59 |  |
| M3XFY0 | PPM1F | 48.46 |  |
| A0A337SSW7 | PSMD1 | 48.34 | 57.71 |
| M3WJ03 | ACTR3 | 48.09 |  |
| M3WPL0 | ITGB1 | 47.95 |  |
| M3XA23 | IPO7 | 47.86 |  |
| A0A337SRS8 | STAT1 | 47.86 |  |
| A0A337SLK8 | RAB1A | 47.50 |  |
| M3X0I4 | MTCH2 | 47.49 |  |
| Q6BCI9 |  | 47.47 |  |
| A0A337SRL7 | TARS1 | 47.43 |  |
| M3WPH3 | TNPO3 | 47.34 |  |
| A0A2I2UZY4 | RANBP2 | 47.28 |  |
| C6K2M7 | GRHPR | 47.28 |  |
| A0A337SCP5 | RPL14 | 47.14 |  |
| M3W8Q7 | GLUD1 | 46.88 |  |
| A0A5F5XJP1 | MYBBP1A | 46.85 |  |
| A0A5F5Y0F6 | LPCAT2 | 46.85 |  |
| M3WMR1 | RPL34 | 46.76 |  |
| A0A337S112 | SHMT1 | 46.75 |  |
| M3VVH1 | PSMD8 | 46.74 | 55.77 |
| A0A5F5Y3V9 | RTL5 | 46.47 | 77.46 |
| M3W6M1 | RPL6 | 46.44 |  |
| A0A337SVT8 | MCM5 | 46.40 |  |
| M3VV51 | PSMC2 | 46.03 | 293.04 |
| A0A337SH53 | EIF3L | 45.85 |  |
| A0A0A0RDA4 |  | 45.33 |  |
| M3WJU8 | RPS26 | 45.30 |  |
| M3VUR5 | STK10 | 45.19 |  |
| M3VZA6 | ETFB | 45.04 |  |
| A0A2I2U4L0 | PAFAH1B1 | 45.00 |  |
| M3XBZ3 | TMCO1 | 44.93 |  |
| M3W059 | RPL13 | 44.90 |  |
| A0A2I2V4B0 | SEC31A | 44.81 |  |
| M3VU96 | ITIH2 | 44.66 |  |
| A0A337SKF9 | MYO5A | 44.59 |  |
| A0A5F5Y033 | SAMHD1 | 44.57 |  |
| M3WJ49 | MCM6 | 44.40 |  |
| A0A5F5XWE4 | MSH2 | 44.25 |  |
| A0A337SRE2 | ARAP1 | 44.23 |  |
| A0A2I2V3N9 | MAP4K4 | 44.05 |  |
| A0A337S5N9 | FCHSD1 | 43.98 |  |
| A0A337SCD2 | ANXA2 | 43.64 |  |
| A0A337S2X5 | YKT6 | 43.61 |  |
| M3WRD0 | AASDHPPT | 43.57 |  |
| A0A2I2UQL4 | RAB21 | 43.44 |  |
| M3W4G4 | WDR77 | 43.34 |  |
| M3WS49 | ATP5PB | 43.13 |  |
| M3WRM0 | RARS1 | 43.12 |  |
| M3VYT4 | IARS2 | 43.03 |  |
| M3XDV7 | DDB1 | 42.99 |  |
| M3WJP6 | HSD17B10 | 42.94 |  |
| A0A2I2V1N2 | DDX21 | 42.94 |  |
| A0A5F5Y2Y4 | DARS1 | 42.81 | 246.44 |
| A0A5F5XH23 | COPG2 | 42.80 |  |
| M3WA00 | NUDT21 | 42.76 |  |
| M3X6G6 | RPL15 | 42.71 |  |
| M3X8Q4 |  | 42.54 |  |
| A0A5F5XLI7 | LOC101080976 | 42.49 |  |
| M3VVV5 | PSMC4 | 42.42 | 132.17 |
| A0A2I2U2M1 | PEPD | 42.32 |  |
| M3W3A3 | ADH5 | 42.28 |  |
| M3WBF7 | CDC37 | 42.25 |  |
| A0A2I2UN91 | SMC2 | 42.17 |  |
| A0A337S539 | SURF4 | 42.09 |  |
| M3W5M8 | PRPF8 | 42.00 |  |
| M3WQ95 | RECQL | 41.82 |  |
| M3W9D5 | LGALS1 | 41.36 |  |
| M3X2D9 | UBA2 | 41.32 |  |
| A0A5F5XJF6 | RPL27A | 41.11 |  |
| A0A2I2UXC0 | MYOF | 40.76 |  |
| A0A5F5Y074 | RUVBL1 | 40.14 |  |
| M3XD21 | ZNF394 | 40.13 |  |
| M3WQ89 | STAM2 | 40.08 |  |
| M3VV10 | PSMD6 | 39.88 |  |
| A0A2I2ULY0 | PYCR2 | 39.60 |  |
| M3XFK8 | TMEM109 | 39.43 |  |
| A0A2I2UCN5 | QARS1 | 39.27 |  |
| M3WDJ1 | MTREX | 38.82 |  |
| M3W9V3 | CACYBP | 38.15 |  |
| A0A2I2UK44 | SNX3 | 37.71 |  |
| M3W0W4 | A1BG | 37.19 |  |
| A0A337SFQ3 | GPI | 36.85 |  |
| A0A5F5Y369 | MERTK | 35.67 |  |
| M3W5Q4 | MRPL3 | 29.75 |  |
| M3WSM7 | LOC101095347 |  | 180.11 |
| Q0QET3 | GAPDH |  | 211.34 |
| A0A2I2UZE5 | PRDX1 |  | 421.99 |
| M3VXR1 | KRT10 |  | 74.06 |
| A0A337SC01 | FN1 |  | 660.05 |
| M3WY56 | CCT6A |  | 213.23 |
| M3WN28 | FGG |  | 612.15 |
| M3WF45 | THBS1 |  | 77.13 |
| Q6ZY26 | APM1 |  | 84.59 |
| M3WSI8 | F2 |  | 65.30 |
| M3VVW0 | BLVRB |  | 144.09 |
| M3WX85 | BPGM |  | 218.06 |
| M3WTN9 | PSMD7 |  | 46.22 |
| A0A5F5Y0K1 | HP |  | 68.25 |
| M3XGD6 | CA1 |  | 146.20 |
